# Impact of steroid therapy on pediatric acute liver failure: prognostic implication and interplay between TNF-α and miR-122

**DOI:** 10.1186/s40348-024-00185-7

**Published:** 2024-12-12

**Authors:** Rania M. El-Shanawany, Eman A. El-Maadawy, Hanaa A. El-Araby, Roba M. Talaat

**Affiliations:** 1 Pediatric Hepatology, Gastroenterology, and Nutrition Department, National Liver Institute, Menoufia University, Menoufia, Shebin El-Koom 32511 Egypt; 2 Molecular Biology Department, Genetic Engineering and Biotechnology Research Institute (GEBRI), University of Sadat City, Sadat City, Egypt

**Keywords:** MicroRNA-122, TNF-α, Acute liver failure, Children, Steroid, Biomarker

## Abstract

**Background:**

Acute liver failure (ALF) is a rare illness marked by rapid deterioration of liver function, leading to high morbidity and mortality rates, particularly in children. While steroids have been observed to correlate with improved survival, evidence supporting their efficacy in ALF children remains limited. miR-122, a liver-specific microRNA, plays a pivotal role in liver pathology, with its expression significantly altered in various liver diseases. Thus, it is considered a potential biomarker for disease progression, aids in prognosis, and identifies therapeutic targets. Our study aims to assess the expression of miR-122 in 24 children with ALF, both before and after steroid therapy, alongside its relationship with tumor necrosis factor-α (TNF-α), to better understand its potential role in treatment response and disease outcomes. miR-122 levels were determined using quantitative real-time RT-PCR (qRT-PCR), while TNF-α levels were assessed using enzyme-linked immunosorbent assay (ELISA) in patient sera.

**Results:**

In ALF children who survived after steroid treatment, miR-122 was markedly decreased compared to both pre-treatment levels (*p* = 0.003) and levels in deceased patients (*p* = 0.01). In addition, TNF-α levels significantly increased in surviving patients compared to pre-treatment levels (*p* = 0.008) and levels in deceased children (*p* = 0.028). A negative correlation was observed between TNF-α and miR-122 following steroids (*r*=-0.46, *p* = 0.04). miR-122 demonstrated 72% sensitivity and 67% specificity in distinguishing survivors and non-survivors, as indicated by its receiver-operated characteristic curve. A positive correlation was found between miR-122 before steroid therapy and both aspartate aminotransferase (AST) and alanine aminotransferase (ALT) before (*r* = 0.641, *p* = 0.002 and *r* = 0.512, *p* = 0.02, respectively) and after (*r* = 0.492, *p* = 0.03 and *r* = 0.652, *p* = 0.003, respectively) steroids treatment.

**Conclusion:**

Our data implies that lower miR-122 levels in steroids-treated ALF children are associated with a better outcome. Although miR-122 is not a strong standalone marker, it could be valuable in a biomarker panel. The increased TNF-α levels and decreased miR-122 expression indicate their involvement in the disease’s pathophysiology. More studies are needed to validate our results.

**Supplementary Information:**

The online version contains supplementary material available at 10.1186/s40348-024-00185-7.

## Introduction

Acute liver failure (ALF) is a rare syndrome characterized by a rapid-onset decline in hepatic function and makes a previously healthy child critically ill with multi-organ failure within days [1]. The cause remains unsettled, and a high mortality rate was associated with this devastating disease [2]. Although steroid treatment has been proposed to mitigate liver damage and improve outcomes, definitive evidence supporting its effectiveness, specifically in children, remains limited [3–5]. Therefore, there is a pressing need for a reliable prognostic marker that can effectively distinguish between survivors and non-survivors following treatment for optimizing patient management. Regretfully, reliable prognostic markers are not yet established for use in children.

Among emerging biomarkers, miR-122 is predominantly expressed in the liver, plays a vital role in regulating hepatocyte function, and is released into circulation upon liver injury [6, 7]. The loss of miR-122 has been implicated in the treatment resistance of hepatocellular carcinoma (HCC) [8] and has been associated with reduced viral load in hepatitis C virus (HCV) infected patients [9]. miR-122 has been reported to be a potential biomarker for diagnosing various liver diseases, such as non-alcoholic fatty liver disease (NAFLD) [10], HCC [11], and drug-induced liver injury [12], and evaluating treatment response in chronic HCV infection [13]. These findings highlight the need to explore miR-122 as a predictive tool and a new therapeutic target in treating ALF in children.

Tumor necrosis factor-α (TNF-α) is a proinflammatory cytokine involved in liver inflammation, fibrosis, and regeneration [14]. The interaction between microRNA and TNF-α represents a crucial dynamic in the progression of various liver diseases [15]. Interestingly, the dysregulation of miR-122 expression has been shown to induce the TNF-α signalling pathway in the liver [16]. Understanding these interactions could provide valuable insights into the pathogenesis of liver diseases and facilitate the development of novel therapeutic strategies targeting these molecular pathways.

Corticosteroids are a class of drugs commonly used to reduce inflammation and are associated with improved survival in ALF patients, as indicated by our previous research [4]. Several studies suggest that miRNA signalling can influence the expression of the drug absorption, distribution, metabolism, and excretion (ADME) genes [17, 18]. Upon exposure to a specific medication, the expression of numerous regulatory miRNAs undergoes significant alteration [19].

Therefore, this study was designed to investigate circulating levels of miR-122 in pediatric ALF patients before and after steroid therapy to elucidate its value in assessing treatment response and disease outcomes. The association between TNF-α and miR-122 expression in response to steroid treatment will be detected to provide valuable insights into the pathophysiology of ALF and aid in refining prognostic algorithms for disease management.

## Subjects and methods

### Study population

A group of twenty-four children with ALF (11 males and 13 females, median age 8.5, ranging from 2 to 16 years) were enrolled in this prospective case-control research. The patients’ pre-steroid therapy (baseline) data served as a control for their post-steroid therapy. Based on the therapeutic outcome, patients were divided into two groups: surviving and deceased groups. Emergent liver transplantation was not considered an option, limiting the outcomes to either survival with native liver or death.

All patients were recruited from the Department of Pediatric Hepatology, Gastroenterology, and Nutrition, National Liver Institute, Menoufia University. The National Liver Institute’s Research Ethics Committee approved this study, which complies with the 1975 Declaration of Helsinki and any subsequent changes (name: NLI IRB 00003413 FWA0000227 and number: 00348). The parents or legal guardians of the included children signed an informed consent form.

ALF inclusion criteria were determined by the sudden development of liver illness without a known history of chronic liver disease, clinical and biochemical evidence of severe liver injury that includes prothrombin time (PT) ≥ 15 s or international normalization ratio (INR) ≥ 1.5 with evidence of hepatic encephalopathy or PT ≥ 20 s or INR ≥ 2 with or without encephalopathy but with coagulopathy that did not improve by vitamin K administration [20].

All patients in the study received either an oral prednisolone dose of 1 mg/kg/day or an equivalent intravenous methylprednisolone dosage of 0.8 mg/kg/day in case of encephalopathy or vomiting, after 12 h of arrival following our established protocol [4]. Steroid withdrawal began after two weeks if PT was below 20 s for conscious patients or below 15 s in patients with encephalopathy. In the deceased group, steroid administration was continued until death. Admission data include age, sex, and any relevant clinical and laboratory data, with follow-up lab results obtained after two doses of steroid therapy. Patients who discontinued steroid treatment (due to uncontrollable side effects) and those who died before completing two steroid doses were excluded from the study. All patients followed the same treatment protocol.

### Blood sampling

Blood samples were collected on sterile tubes containing ethylenediaminetetraacetic acid (EDTA). The tubes were centrifuged, and the plasma was carefully collected and stored at -20 C for subsequent analysis of miR-122 and TNF-α.

### RNA extraction and quantification of miRNA-122 expression level

Total RNA was purified from plasma samples (200𝜇l) using miRNeasy Mini kit (Qiagen, Valencia, CA, USA; catalog number 217004) according to the manufacturer’s directions. The purity and concentration of RNA were spectrophotometrically evaluated by NanoDrop™ 2000/2000c (Thermo Fisher Scientific, Waltham, MA, USA). A total of 100 ng RNA was reverse transcribed using miScriptII RT kit (Qiagen, catalog number 218061) according to the manufacturer’s guidelines.

Quantitative real-time PCR (qRT-PCR) for Hsa_miR-122 was carried out in 25 µL PCR reactions using miScript Primer Assays (Qiagen) and miScript SYBR Green PCR kit (catalog number 218073). The PCR cycles were as follows: 95 °C for 15 min, followed by 40 cycles of 95 °C for 15 s, 55 °C for 30 s, and 70 °C for 30 s. A post-amplification melting curve program was run at 95 °C for 30 s, 65 °C for 30 s, and 95 °C for 30 s.

The U6B small nuclear RNA (RNU6B) expression was used as an endogenous control for data normalization. All experiments were performed in AriaMx Real-Time PCR System (Agilent Technologies, USA). Differences in the cycle threshold (Ct) values between the tested miRNA and reference genes were calculated to determine the relative expression levels using the 2^−ΔΔCt^ method with data analysis center of Qiagen (https://geneglobe.qiagen.com/eg/analyze).

### TNF-α plasma level assessment

The commercial enzyme-linked immunosorbent assay (ELISA) kit (Innova Biotechnology Co., Ltd.) was used to measure the levels of TNF-α in the patient’s plasma according to the manufacturer’s instructions. The optical absorbance of each well at 450 nm was determined using a microplate reader (SunriseTM, Tecan Group Ltd. Manndorf, Switzerland).

The standard curve produced by the ELISA reader-control software (Softmax; Molecular Devices, Sunnyvale, CA, USA) utilizing digital data of a raw absorbency value was used to instantly calculate the cytokine content of unidentified samples. The data were expressed in picograms of cytokine per milliliter of plasma (pg/mL).

### Statistical analysis

SPSS program 21 (Statistical Package for Social Science, IBM Corporation, USA) was used to perform all statistical analyses. Given the limited sample size, data was represented non-parametrically by the median, minimum, and maximum values, while categorical data was represented using proportions. The Mann-Whitney *U* test was applied to compare two research groups, whereas the Kruskal-Wallis test was used to compare several groups. The Wilcoxon signed-rank test assessed differences in laboratory data before and after treatment. For qualitative data, significance was measured using the Chi-square or the Fisher exact test. Spearman was used to test the correlation. All tests were two-sided, with a significance level set at p˂ 0.05. A receiver-operated characteristic (ROC) curve was employed to analyze the scoring system’s sensitivity versus false positive specificity. Together with ROC analysis, the Youden Index (https://www.mdapp.co/youden-index-calculator) was utilized to assess the performance of a diagnostic test.

## Results

### Patient’s characteristics

Table (1) displays the clinical data for the recruited ALF patients. The survival rate after therapy was 54.2%. Table (2) presents the laboratory data for all ALF patients before and after steroid therapy. After steroid treatment, children showed an improvement in PT (*p* = 0.01) and INR (*p* = 0.01) compared to their results before treatment, in addition to a significant improvement in liver enzymes as aspartate aminotransferase (AST) (*p* < 0.001), alanine aminotransferase (ALT) (*p* = 0.009), and alkaline phosphatase (ALP) (*p* = 0.01), compared to their tests before starting steroids therapy. When comparing patients based on their outcome (deceased vs. surviving) after treatment, Table (3) indicates a significant improvement in all tested coagulation parameters (PT, INR, and PTT). Historical data of 25 ALF children (11 males and 14 females, median age 5, ranging from 1.4 to 14 years) from our center before the introduction of steroid therapy was collected and revealed a 24% survival rate and a 76% mortality rate (Table 4). Supplementary Table (1) illustrates the etiology and laboratory data of the historical ALF patients who did not receive steroids. Supplementary Table (2a-2b) indicates no significant difference in the mortality rate across different etiologies in both groups.

**Table 1:** Clinical findings of the studied acute liver failure (ALF) patients

Clinical data	ALF group (*N* = 24)
**MAP (mmHg)**	76 (63–93)
**Encephalopathy**	15 (62.5%)
**Hepatomegaly**	12 (50.0%)
**Splenomegaly**	10 (41.7%)
**Ascites**	3 (12.5%)
**Complication**	
• Sepsis	18 (75.0%)
• Renal impairment	10 (41.7%)
• Pneumonia	6 (25.0%)
• Circulatory failure	7 (29.2%)
**Etiology**	
• Indeterminate	14 (58.3%)
• HAV	9 (37.5%)
• Wilson	1 (4.2%)

Data are presented as median, minimum, maximum, and percentage. *MAP* Mean arterial pressure in millimeters of mercury (mmHg), *HAV* hepatitis A virus

**Table 2:** Laboratory findings of all ALF patients before and after steroid therapy

**Investigated items**	**Before treatment** **(*N*=24)**	**After treatment** **(*N*=24)**	***P*-value**
**Haematological data**			
Haemoglobin (g/dl)	10.6 (6-15.5)	10.8 (8.1-14.4)	0.64
WBCs (10^3^/µL)	9.6 (3.1-21)	14.0 (2.6-20.5)	**0.03**
Platelets (10^3^/µL)	313 (121-600)	325 (119-756)	0.23
**Coagulation data**			
PT (seconds)	37 (26-60)	30 (18-60)	**0.01**
INR	3.5 (2.4-6.0)	2.7 (1.6-6)	**0.01**
PTT (seconds)	69 (52-120)	66 (38-120)	0.06
**Biochemical data**			
AST (U/L)	807 (110-4133)	537 (93-1970)	˂ **0.001**
ALT (U/L)	1098 (19-3141)	729 (10-2393)	**0.009**
Albumin (g/dl)	3.4 (1.7-4.2)	3.5 (1.8-4.3)	0.38
ALP (U/L)	284 (15-575)	214 (35-547)	**0.01**
GGT (U/L)	49 (11-295)	53 (13-180)	0.38
Total bilirubin (mg/dl)	20 (5-52)	19 (5-60)	0.98
Direct bilirubin (mg/dl)	13 (4-29)	13 (4-39)	0.67
Urea (mg/dl)	15 (2-59)	15 (8-104)	0.18
Creatinine (mg/dl)	0.4 (0.16-1.3)	0.42 (0.12-1.45)	0.17
CRP (mg/dl)	2.3 (0.2-23)	5.0 (1-18)	0.19

All data are presented as median, minimum, and maximum. *AST* Aspartate aminotransferase, *ALT* Alanine aminotransferase, *ALP* Alkaline phosphatase, *GGT* Gamma-glutamyl transferase, *PT* Prothrombin time, *INR* International normalized ratio, *WBCs* White blood cells, *CRP* C-reactive protein, *mg/dl* Milligrams per decilitre, *U/L* International units per liter, *g/dl* Grams per liter, *µL* Microliter

**Table 3:** Laboratory findings of deceased and surviving ALF patients after steroid therapy

**Investigated items**	**Deceased ALF patients** **(*N*=11)**	**Surviving ALF patients** **(*N*=13)**	***P*-value**
**Haematological data**			
Haemoglobin (g/dl)	12 (8.6-14)	10.7 (8.1-14.4)	0.33
WBCs (10^3^/µL)	15 (2.6-20.5)	13 (4.3-20)	0.90
Platelets (10^3^/µL)	325 (119-756)	336 (125-599)	0.70
**Coagulation data**			
PT (seconds)	44 (30-60)	25 (18-44)	˂ **0.001**
INR	4.1(2.8-6)	2.2 (1.6-4)	˂ **0.001**
PTT (seconds)	89 (61-120)	57 (38-88)	˂ **0.001**
**Biochemical data**			
AST (U/L)	448 (134-1800)	590 (93-1970)	0.30
ALT (U/L)	681 (10-1871)	973 (47-1773)	0.44
Albumin (g/dl)	3.4 (1.8-4.2)	3.6 (2.9-4.3)	0.66
ALP (U/L)	201 (35-547)	259 (127-508)	0.61
GGT (U/L)	38 (29-79)	73 (13-180)	0.24
Total bilirubin (mg/dl)	23.5 (12-60)	18 (5-47)	0.08
Direct bilirubin (mg/dl)	16 (5-39)	10 (4-26)	0.26
Urea (mg/dl)	17 (8-104)	14 (8-36)	0.34
Creatinine (mg/dl)	0.69 (0.39-1.45)	0.35 (0.12-0.82)	**0.003**
CRP (mg/dl)	1.8 (1-5.5)	6 (3.8-18)	**0.03**

All data are presented as median, minimum, and maximum. *AST* Aspartate aminotransferase, *ALT* Alanine aminotransferase, *ALP* Alkaline phosphatase, *GGT* Gamma-glutamyl transferase, *PT* Prothrombin time, *INR* International normalized ratio, *WBCs* White blood cells, *CRP* C-reactive protein, *mg/dl* Milligrams per decilitre, *U/L* International units per liter, *g/dl* Grams per liter, *µL* Microliter

**Table 4:** The outcome of steroid treatment in ALF patients

**Characteristics**	**Historical group** **(Did not receive steroids)** **(*N*=25)**	**Current study group (Received steroids)** **(*N*=24)**	***P*-value**
**Duration of steroids**			
Median (days) (minimum-maximum)	--	18.5 (9 -45)	
**Outcome**			
• Survived	6 (24%)	13 (54.2%)	**0.03**
• Deceased	19 (76%)	11 (45.8%)	

### miR-122 expression level

The expression of miR-122 in all patients of the ALF group and subgroups was demonstrated in Table (5). A significant reduction in miR-122 levels was observed in children after steroid treatment compared to before treatment (*p* = 0.03). Concerning therapy outcome, the surviving ALF children showed significant downregulation of miR-122 expression compared to deceased ones (*p* = 0.01) after steroid therapy. These interesting results were further supported by our findings that the surviving children showed a significant decrease (*p* = 0.003) in miR-122 levels compared to their levels before steroid therapy. An insignificant difference was observed in miR-122 expression in the deceased children before and after therapy. No significant difference in miR-122 level was observed among different etiologies both before and after steroid therapy (Table 6).

**Table 5:** Plasma level of microRNA-122 and TNF-α before and after steroid therapy in the studied groups

**Parameters**	**All ALF patients (before starting steroid)** **(*N*=24)**	**All ALF patients (after starting steroid)** **(*N*=24)**	**Deceased ALF patients** **(*N*=11)**		**Surviving ALF patients** **(*N*=13)**	
			**Before starting steroid**	**After starting steroid**	**Before starting steroid**	**After starting steroid**
**miR-122** **(RCF)**	0.99 (0.02-5.86)	0.28^a^^*^ (0.02-2.51)	0.27 (0.02-4.14)	0.84^b*^ (0.2-4.51)	1.96 (0.02-5.86)	0.06^c**^ (0.02-1.71)
**TNF-α** **(pg/ml)**	227.9 (165.5-391.7)	303.6 (140.7-593.6)	220.6 (165.5-391.7)	215.8^b*^ (140.7-529.4)	254 (201-356)	312^c**^ (199-593)

All data are presented as median, minimum, and maximum. a: significance between before and after starting steroids for all patients. b: significance between the post-starting steroid groups (deceased and surviving). c: significance between before and after starting steroid for the surviving ALF group. *: *P*-value ˂0.05; **: *P*-value ˂0.01. RFG: relative fold change

**Table 6:** Plasma levels of microRNA-122 and TNF-α before and after steroid therapy among the different ALF etiologies

**Parameters**	**HAV** **(*N*=9)**	**Indeterminate** **(*N*=14)**	**Wilson** **(*N*=1)**	***P*-value**
**Before starting steroid therapy**				
**miR-122** **(RFC)**	2.17 (0.02-5.86)	0.38 (0.02-4.14)	1.96 (1.96-1.96)	0.41
**TNF-α** **(pg/ml)**	254 (204-295.6)	227.1 (165.5-391.7)	220.6 (220.6-220.6)	0.61
**After starting steroid therapy**				
**miR-122**	0.42 (0.02-1.27)	0.23 (0.02-4.51)	0.85 (0.85-0.85)	0.90
**TNF-α** **(pg/ml)**	312.2 (140.7-593.6)	259.4 (199.4-387.7)	202.7 (202.7-202.7)	0.39

All data are presented as median, minimum, and maximum; *pg/ml* Picograms per milliliter. HAV hepatitis A virus infection. *RFG* relative fold change

**Fig. 1:**
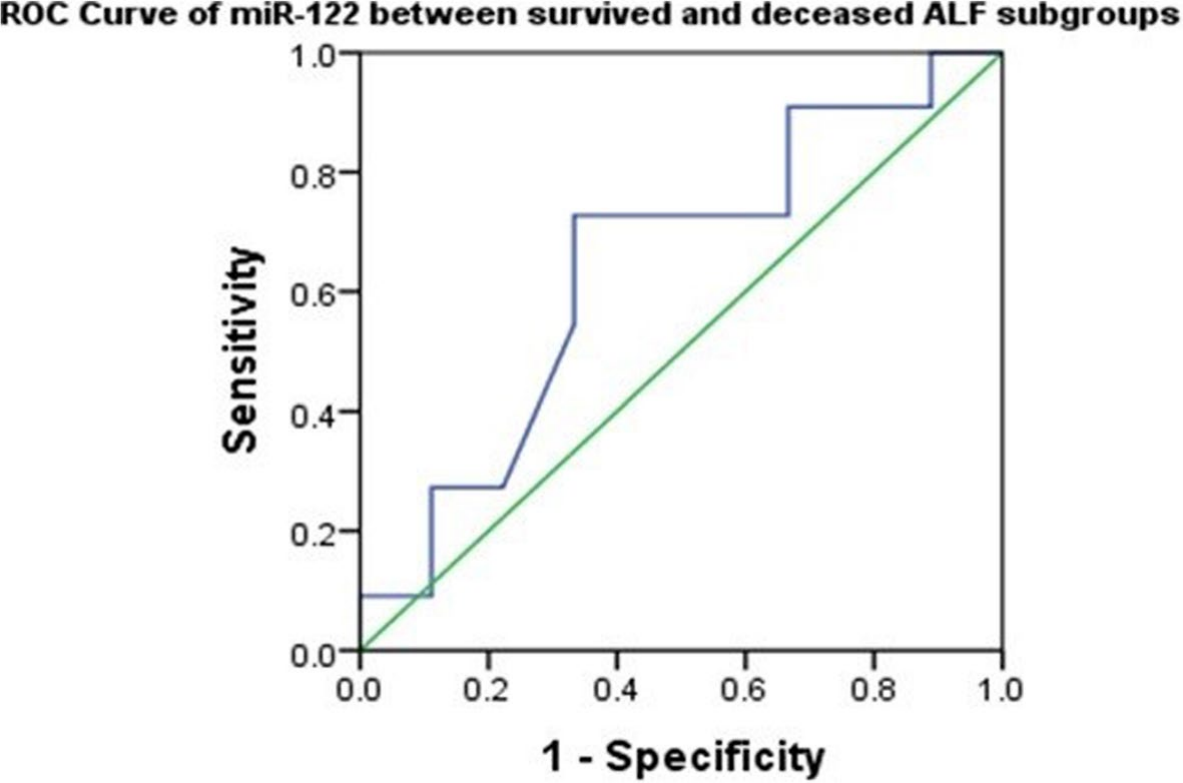
Roc curve of miR-122. When discriminating survivors from non-survivors, miR-122 exhibits a sensitivity of 72% and specificity of 67% at a cut-off value of 0.507

ROC curve (Fig. 1) showed a sensitivity of 72% and specificity of 67% at a cut-off value of 0.507 (with Youden Index = 0.39), and the area under the curve was 0.641 (95% CI: 0.388–0.859; *p* = 0.287). Although the results showed that miR-122 is not a strong predictor for an outcome, it could contribute as a part of a multi-biomarker panel in discriminating between surviving and deceased ALF patients.

### Plasma level of TNF-α

Regarding therapy outcome, the TNF-α levels in the surviving group were significantly elevated (*p* = 0.008) after steroid therapy compared to before. Moreover, after steroid treatment, surviving ALF patients showed a significant increase in TNF-α levels compared to deceased ALF patients (*p* = 0.028) (Table 5). There was no significant difference in TNF-α level between different etiologies before or after steroid therapy (Table 6).

### Correlation of miR-122, TNF-α, and laboratory findings

**Fig. 2:**
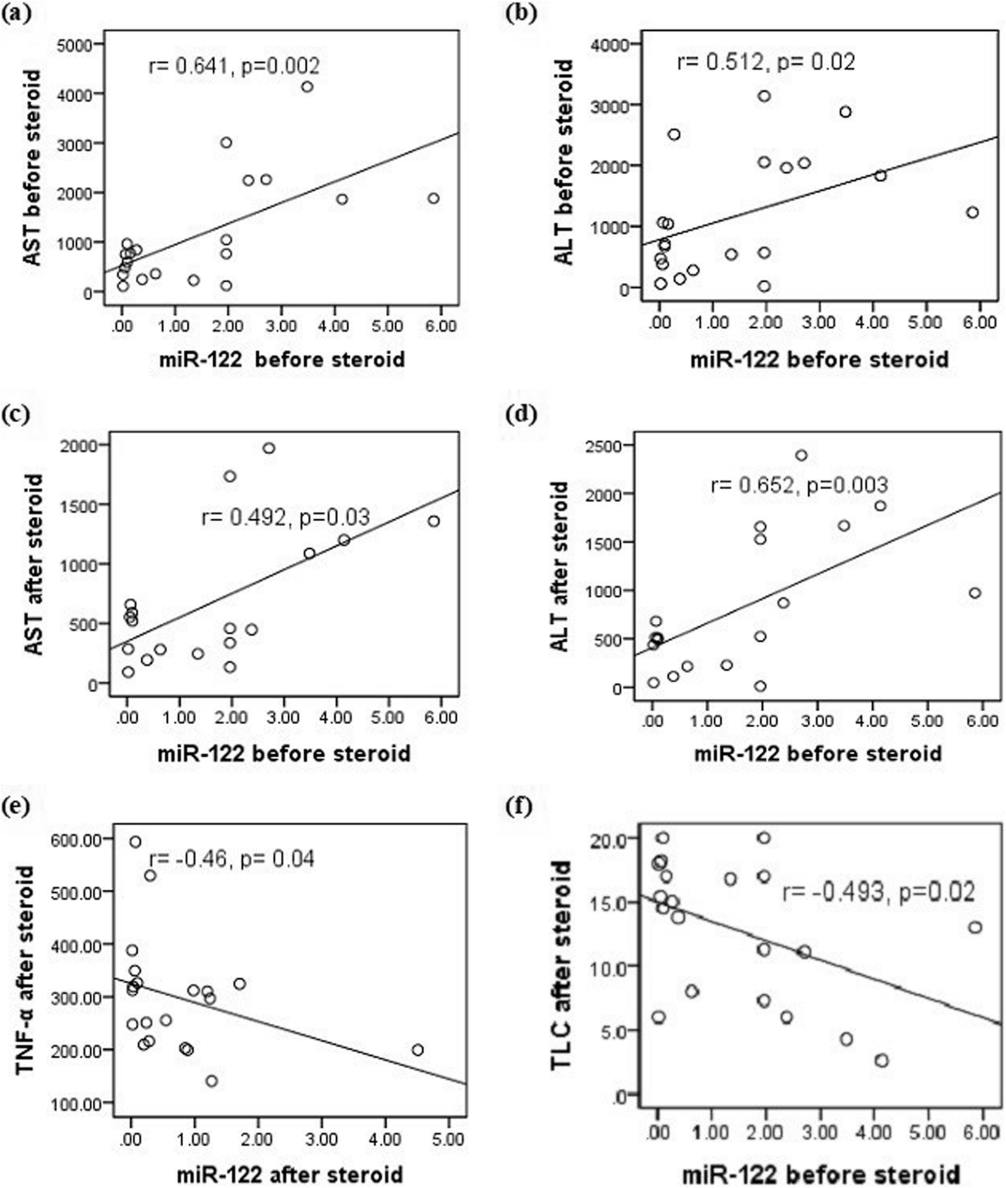
Correlation of miR-122 with different parameters. In ALF patients, miR-122 expression level before steroids significantly correlates with AST and ALT plasma levels before (2a, 2b) and after steroid therapy. miR-122 positively correlates with TLC after treatment. miR-122 negatively correlates with TNF-α levels after steroid therapy. ALT: Alanine aminotransferase; AST: Aspartate aminotransferase; TLC: Total leucocyte count

miR-122 expression level before steroid therapy exhibited a significant positive correlation with AST and ALT plasma levels either before (*r* = 0.641, *p* = 0.002 and *r* = 0.512, *p* = 0.02, respectively) or after (*r* = 0.492, *p* = 0.03 and *r* = 0.652, *p* = 0.003, respectively) steroids therapy. miR-122 positively correlates (*r* = 0.493, *p* = 0.02) with total leucocyte count (TLC) after treatment. On the other hand, miR-122 expression level negatively correlated with TNF-α plasma level (*r*=-0.46, *p* = 0.04) after steroid therapy (Fig. 2).

### Association of miR-122 and TNF-α with disease clinical manifestation

As demonstrated in Table (7), no statistical significant difference was observed in miR-122 or TNF-α and ALF clinical manifestations.

**Table 7:** Association between TNF-α and mir-122 levels with clinical manifestation in ALF children

**Investigated items**	**ALF patients** **(*N*=24)**	
	**TNF-α**	**miR-122**
**Sex**		
Female	220 (165-281.9)	1.34 (0.07-4.14)
Male	288 (196.1-391.7)	0.63 (0.02-5.86)
**Encephalopathy**		
Yes	235 (165.5-391.7)	0.32 (0.02-4.14)
No	271 (204.3-356.7)	1.96 (0.02-5.86)
**Hepatomegaly**		
Yes	254.7 (204.3-391.7)	1.17 (0.02-3.48)
No	223.8 (165.5-356.7)	0.99 (0.06-5.86)
**Splenomegaly**		
Yes	271.7 (204.3-391.7)	1.29 (0.02-3.48)
No	223.8 (165.5-295.6)	0.86 (0.06-5.86)
**Ascites**		
Yes	212.4 (204.3-220.6)	0.99 (0.02-1.96)
No	254.7 (165.5-391.7)	0.99 (0.02-5.86)
**Complication**		
** Sepsis**		
Yes	220.6 (165.5-391.7)	0.38 (0.02-5.86)
No	255.5 (254-300.2)	1.96 (0.10-3.48)
** Renal failure**		
Yes	217.3 (165.5-281.9)	0.21 (0.06-2.38)
No	255.5 (204.3-391.7)	1.96 (0.02-5.86)
** Pneumonia**		
Yes	243 (214.1-391.7)	0.38 (0.02-4.14)
No	254 (165.5-356.7)	1.34 (0.02-5.85)
** Circulatory failure**		
Yes	243 (214.1-281.9)	0.38 (0.07-4.14)
No	254 (165.5-391.7)	1.34 (0.02-5.86)

## Discussion

ALF is a disease with different etiologies [21]. Despite advancements in treatment methods, including liver transplantation [22] and steroid therapy [5], predicting outcomes in ALF remains challenging. Discriminating survivors from non-survivors within the ALF group is crucial for clinical decision-making after receiving the steroid therapy. miR-122 is the most abundant microRNA in liver cells [23]. To fill this knowledge gap, we subdivided the ALF group into surviving and deceased groups, measuring the expression levels of miR-122 and TNF-α before and after steroid therapy. This approach aims to better understand the relationship between corticosteroid treatment and ALF outcomes, offering potential insight into the role of miR-122 as a non-invasive survival biomarker and a possible therapeutic target.

Our study showed that, after steroid therapy, miR-122 expression was significantly downregulated in surviving patients compared to deceased ones. Moreover, miR-122 levels in survivors were much lower after steroid treatment than at baseline. Several assumptions were proposed to explain the change in miR-122 expression in response to steroids. Arora et al. [24] revealed that drugs could influence microRNA expression, while Clayton et al. [25] demonstrated that the beneficial effects of glucocorticoids may be enhanced by targeting specific microRNA expression, mediating different cell actions. Moreover, miRNAs can indirectly influence the responsiveness of cells to glucocorticoids via modifying glucocorticoid receptor expression in myeloma cells [26]. Hu et al. [27] further demonstrated that miR-122 inhibition reduces oxidative stress and inflammation in mice with NAFLD. These findings suggest that the observed decrease in miR-122 expression in survivors may reflect a beneficial effect of steroids on liver function in ALF patients.

On the other hand, John et al. [28] demonstrated increased miR-122 levels in serum and hepatocytes during liver regeneration in spontaneous recovery from ALF. A combination of factors, such as differences in demographics, comorbidities, and genetic variability between participants in both studies could explain the divergence of results. Additionally, the timing of measurements of miR-122, taken earlier in the course of illness, and variation in treatment protocol could also contribute to the contrasting results.

Focusing on biochemical parameters, ALT and AST are enzymes predominantly found in hepatocytes, and their serum levels serve as standard biomarkers for hepatocellular injury [17]. Our study found a correlation between miR-122 levels and markers of hepatocyte injury, specifically ALT and AST. The results indicated that miR-122 levels were elevated shortly after hepatocyte injury and exhibited a positive correlation with both AST and ALT levels before and after steroid therapy. Starckx et al. [29] reported similar results in rats with acute liver injury induced by different liver toxins.

The correlation between TNF-α and miR-122 in the liver appears multifaceted, as their interplay contributes to the pathogenesis of many liver diseases. Our findings indicate a negative correlation between TNF-α and miR-122. The increase in TNF-α levels is associated with the downregulation of miR-122 in patients with ALF. Although the exact mechanism is not fully defined, miR-122 expression might modulate TNF-α and affect its function in the liver failure group. Previous Studies have explored this relationship. Li et al. [16] showed that TNF-α could downregulate miR-122 expression level in HCC in mice. Similarly, Hsu et al. [30] found that miR-122 deletion showed increased TNF-α production in both mice and cell lines in response to lipopolysaccharide-induced inflammation. Shi et al. [15] obtained the same results in hepatoma cells. miR-122 has been shown to target the serine-threonine protein kinase (PKR) activator, which leads to the generation of nuclear factor kappa B (NF-κB) and may prevent the production of proinflammatory cytokines [31]. Consequently, we can assume that the increased levels of TNF-α coincide with decreased levels of miR-122, potentially affecting NF-κB expression and thereby enhancing the chance of survival in children with ALF.

One of the study’s limitations is the heterogeneity in the etiologies among ALF patients’ cohort. While we analyze the data by stratifying according to different disease etiologies, diversity remains a factor that could affect the results interpretation. Future research with a more homogenous patient population or a larger sample size across specific etiology may help to clarify these effects. In addition, investigations are warranted into the correlation between hepatocyte damage reversibility and miR-122 levels on histopathological bases. Until now, we know very little about how miR-122 influences the treatment response in liver damage, so further studies may refine its therapeutic value and application in personalized medicine for liver diseases.

## Conclusion

Our study found that, in ALF children, miR-122 levels were lower in steroids-treated patients who survived, indicating a potential association between miR-122 and patient outcomes. Further research is needed to better understand the role of miR-122 in treatment response and disease outcome. Although miR-122 alone did not show strong predictive performance, it is recommended to measure its plasma level as a part of the biomarkers panel of steroids-treated ALF children. Additionally, the observed increase in TNF-α level, alongside a concurrent decrease in miR-122 expression, points to their potential roles in the pathophysiology of this severe illness, where miR-122 may help modulate TNF-α mediated immune cell responses. Consequently, this highlights the need for further investigation to explore how miR-122 and TNF-α may contribute to immune cell profiles observed in ALF.

## Supplementary Information

Supplementary Material 1.

## Data Availability

No datasets were generated or analysed during the current study.
