# Supplementary material for: Impact of steroid therapy on pediatric acute liver failure: prognostic implication and interplay between TNF-α and miR-122
Source: Mol Cell Pediatr. 2024 Dec 12;11:13. doi: 10.1186/s40348-024-00185-7 (PMC11638456; doi:10.1186/s40348-024-00185-7)
Supplement: Supplementary file 1 — Supplementary Material 1. [file 40348_2024_185_MOESM1_ESM.docx]

**Supplementary Table 1:** Etiology and laboratory data of the historical ALF patients who did not receive steroids

| **Investigated items** | **Historical ALF patients**  **(Did not receive steroids)**  **(N=25)** |
| --- | --- |
| **Etiology**   - Indeterminate - HAV - Wilson | 13 (52%)  10 (40%)  2 (8%) |
| **Haematological data** | |
| Haemoglobin (g/dl) | 9 (3.3-13.5) |
| WBCs (10^3^/µL) | 11 (0.7-27) |
| Platelets (10^3^/µL) | 217 (19-745) |
| **Coagulation data** | |
| PT (seconds) | 50 (17-90) |
| INR | 4.3 (1.5-7) |
| PTT (seconds) | 67 (29-140) |
| **Biochemical data** | |
| AST (U/L) | 511 (148-4583) |
| ALT (U/L) | 672 (15-3608) |
| Albumin (g/dl) | 3 (1.9-4) |
| ALP (U/L) | 290 (3-1720) |
| GGT (U/L) | 68 (21-310) |
| Total bilirubin (mg/dl) | 22 (3-52) |
| Direct bilirubin (mg/dl) | 13 (1.8-39) |
| Urea (mg/dl) | 19 (5-240) |
| Creatinine (mg/dl) | 0.5 (0.11-4.2) |
| CRP (mg/dl) | 19 (0.7-42) |
| All data are presented as median, minimum, and maximum. AST: Aspartate aminotransferase; ALT: Alanine aminotransferase; ALP: Alkaline phosphatase; GGT: Gamma-glutamyl transferase; PT: Prothrombin time; INR: International normalized ratio; WBCs: White blood cells; CRP: C-reactive protein; mg/dl: Milligrams per deciliter; U/L: International units per liter; g/dl: Grams per liter; µL: Microliter. | |

**Supplementary Table 2a:** Outcome of different etiologies in Current study group

| **Parameters** | **HAV**  **(N=9)** | **Indeterminate**  **(N=14)** | **Wilson**  **(N=1)** | ***P*-value** |
| --- | --- | --- | --- | --- |
| **Outcome**   - **Deceased** - **Survived** | 3 (33.3%)  6 (66.7%) | 7 (50%)  7 (50%) | 1(100%)  -- | 0.52 |

**Supplementary Table 2b:** Outcome of different etiologies in historical group

| **Parameters** | **HAV**  **(N=10)** | **Indeterminate**  **(N=13)** | **Wilson**  **(N=2)** | ***P*-value** |
| --- | --- | --- | --- | --- |
| **Outcome**   - **Deceased** - **Survived** | 7 (70%)  3 (30%) | 10 (76.9%)  3 (23.1%) | 2 (100%)  -- | 0.65 |
